# Anti-obesity effects of yuja (*Citrus junos* Sieb ex Tanaka) pomace extract fermented with lactic acid bacteria on the hepatocytes and epididymal fat tissue of rats

**DOI:** 10.1186/s42649-023-00090-9

**Published:** 2023-08-09

**Authors:** Han-Na Chu, Haeng-Ran Kim, Kyeong-A. Jang, Yu-Jin Hwang, Jeong-Sang Kim

**Affiliations:** 1 Department of Agro-Food Resources, National Institute of Agricultural Sciences, Wanju, 55365 Korea; 2 College of Korean Medicine, Dongshin University, Naju, 58245 Korea

**Keywords:** Yuja pomace, *Citrus junos* Sieb ex Tanaka, Anti-obesity, Fermentation, Lactic acid bacteria, Hepatocytes, Epididymal fat cell

## Abstract

This study investigated the anti-obesity effects of yuja pomace extract fermented with lactic acid bacteria in rats with high-fat diet-induced obesity over a period of eight weeks. Epididymal fat cell size was significantly smaller, by about 33%, in the treatment groups given yuja pomace extract fermented with lactic acid bacteria compared to the CON group. Electron microscopic observation of hepatocyte microstructure showed that the number of lipid droplets was lower in hepatocytes, the number of mitochondria was higher, along with distinct cristae, and the rough endoplasmic reticula were well developed with stacks of cisternae and ribosomes. Thus, it is believed that yuja pomace extract fermented with lactic acid bacteria, by influencing body weight and lipid accumulation, is effective in the prevention and treatment of obesity.

## Introduction

Obesity rates have been rapidly increasing in Western countries, including the United States, for decades. Not only adult obesity, but childhood obesity is increasing as well. This has led the health authorities of several countries to conduct numerous studies in search of countermeasures and preventive measures (Karam and McFarlane 2007). Yuja (*Citrus junos* Sieb ex Tanaka) is the fruit of the yuja tree. In Dongeuibogam, it is stated that “Yuja has thick skin, tastes sweet, and has no toxins. It weakens bad ‘qi’ in the stomach, treats alcohol poisoning, and eliminates the foul breath of drunken men.” Yuja is rich in vitamins A and C and limonene, and also contains dietary fiber, pectin, and limonoid compounds, which is why it has been widely known as beneficial to human health (Kim 1999).Unlike other citrus fruits, both the pulp and skin of yuja, which has a unique sourness and flavor, are consumed, and it is commonly used to make yuja-cheong (yuja honey), which is a sugaring product of yuja, and yuja juice, extracted from yuja pulp (Yang and Eun 2011). But consuming, directly or diluted, yuja leached with a large amount of sugar can cause metabolic disorders or carbohydrate metabolism-associated problems, and the leaching process takes a long time, causing time-related problems (Ryu and Kwon 2012). Yuja honey made with both the pulp and skin of yuja is the most common yuja product, as it produces the smallest amount of byproducts and wastes. Recently, however, more products are being manufactured through a variety of processing methods, producing byproducts such as yuja skin, seed, and pulp (Lee et al. 2017; Lim et al. 2012; Yoon et al. 2016). To solve this issue, it seems necessary to develop functional products made with yuja pomace, which is discarded after processing. According to recent studies, anti-obesity effects were proven through the expression of peroxisome proliferator-activated receptor gamma (PPAR-γ) and AMP-activated protein kinase (AMPK) in the skin of yuja (Kim et al. 2013), which is rich in unsaturated fatty acids. In addition, the regulatory effect on leptin secretion, which has an anti-obesity effect and suppresses appetite, was observed by analyzing yuja seed oil extract (Kim et al. 2014). It is therefore necessary to develop products that can supplement the weaknesses of using yuja pomace and maximize its functional components, based on the anti-obesity effects of yuja skin, seeds, and some pulp used in yuja pomace shown in previous studies. Because yuja pomace contains plenty of fiber, such as pectin, fermentation can degrade cell walls during processing. Although fermentation can be done using degrading enzymes that are widely available, there are time and economic limitations involved. An efficient fermentation method was thus suggested, involving the degradation of the fiber in yuja pomace using helpful microbes (Doh et al. 2010), among which lactic acid bacteria are anaerobes known to destroy plant cell walls (Flint and Bayer 2008), detoxify raw materials, and help extract active ingredients during fermentation (Katiyar 2007). Fermentation with lactic acid bacteria has been studied by many investigators using various materials. However, there are not many preliminary studies to prove anti-obesity effects using fermentation with lactic acid bacteria, thus it is thought that more studies are needed in this area. The author of a previous study manufactured yuja extract fermented with lactic acid bacteria using *Lactobacillus plantarum* on yuja pomace, which was then fed to rats with diet-induced obesity to confirm the inhibitory effect on fat accumulation, and also examined the functional value of food byproducts and the safety of fermentation with lactic acid bacteria (Chu and Kim 2018). Thus, this study was performed to investigate the utilization of yuja pomace as a high-functionality waste resource and the anti-obesity effects of yuja pomace extract fermented with lactic acid bacteria in rats with diet-induced obesity.

## Materials and Methods

### Experimental animals

Six-week-old, male Sprague–Dawley rats, weighing around 180 g each, were purchased and kept for one week, for the purpose of acclimation before the experiment, in the animal breeding room of the College of Korean Medicine of Dongshin University. The animal room was kept at a lighting level of 150 to 300 Lux, temperature of 21 ± 2℃, and humidity of 50 to 60% and subjected to a 12-h light–dark cycle. During the experimental period, all animals were provided with solid feed, 60% high-fat feed, and drinking water ad libitum. All experiments were performed after the approval of Dongshin University Institutional Animal Care and Use Committee (IACUC) (Approval No.: DSU2016-10–02) (Chu and Kim 2018).

### Enzyme-hydrolyzed yuja pomace extract and yuja pomace extract fermented with lactic acid bacteria

The enzyme-hydrolyzed yuja pomace extract was obtained using commercial enzymes (Novozymes Co., Denmark) generally used in food manufacturing processes. Purified water (30%) was added to 1 kg of yuja pomace and then heat sterilized at 75℃ for 10 min. The ratio of added enzymes was 4:1:2 of cellulase (celluclast), pectinase (cirtozyme), and amyloglycosidase (AMG), respectively, which was determined on the basis of preliminary experiments. A 2% enzyme mix was added and hydrolyzed at 45℃ for five hours, after which residues were eliminated by filter press to obtain enzyme-hydrolyzed yuja pomace extract. For the separation of lactic acid bacteria, traditional yeast was purchased at stores in the Jeollanam-do area, suspended in 1% lactic acid solution, and then pulverized, filtered, and diluted to appropriate concentrations. The diluted solution was smeared onto Lactobacilli MRS agar (Difco Co., France) medium and incubated at 37℃ for 24 h. After incubation, a streak inoculation with the colony formed was made on the MRS media with 2% CaCO3. Strains with a clear zone at least 5 mm away from the strain body were sorted out first. These strains were streak-inoculated onto the CMC agar (20 g/L carboxymethyl cellulose, 2.5 g/L yeast extract, 5 g/L K2HPO4, 1 g/L NaCl, 0.2 g/L MgSO4. 7H2O, 0.6 g/L (NH4)2SO4, and 15 g/L agar) medium and incubated at 37℃ for 48 h. After incubation, it was stained with Gram's iodine solution, in which 2% I2 and 3% KI were dissolved in 70% ethanol, or 1% Congo-red, and then strains with a yellow-colored clear zone were finally selected. As a result, a total of about 10,000 active lactic acid bacteria were separated at first, and then 90 lactic acid bacteria with cellulolytic activity were separated. Among these, 10 strains with excellent carboxymethylcellulase (CMCase) activity were separated, and then the final five lactic acid bacteria with excellent yuja pomace-degrading efficiency were separated and selected as GAVOL-06(‘Gavofarms’ + *’Lactobacillus plantarum’*-06) (79.4%, A6 group), GAVOL-07 (68.9%, A7 group), GAVOL-08 (75.0%, A8 group), GAVOL-09 (67.8%, A9 group), and GAVOL-10 (73.3%, A10 group). The extracts fermented with lactic acid bacteria were manufactured by adding novel fiber-degradable lactic acid bacteria, *Lactobacillus plantarum* GAVOL-6 to -10. Purified water (20%) was added to 1 kg of yuja pomace and heat sterilized at 70℃ for one hour to eliminate any contaminants in the yuja pomace, before the inoculation of the starters for lactic acid bacteria fermentation. *Lactobacillus plantarum* GAVOL-6 to -10, as starters, were diluted with physiological saline, and 8.0 log cfu/ml was inoculated and incubated at 37℃ for 48 h. Residues were eliminated by filter press to obtain yuja pomace extract fermented with lactic acid bacteria.

### Administration of the fermented extract

Yuja pomace extract fermented with lactic acid bacteria was mixed into drinking water and administered to the treatment groups for eight weeks. The animals were divided into eight groups, with seven animals per group, as follows: normal diet group (NOR) given general solid feed, control group (CON) fed a 60% high-fat diet (HFD), treatment group (A0) fed a 60% HFD and 2 mL/kg/day enzyme-hydrolyzed yuja pomace extract, and treatment groups (A6, A7, A8, A9, and A10) given a 60% HFD and 2 mL/kg/day yuja pomace extract fermented with lactic acid bacteria.

### Body weight measurement

Body weight was measured down to the gram using a digital scale (TS4KK, OHAUS Corporation, Parsippany, NJ, USA) once a week during the experimental period (Chu and Kim 2018).

### Relative weight of liver and epididymal fat

Liver and epididymal fat were dissected out, rinsed with PBS, and weighed using a balance (WB0710037, KERN&Sohn GmbH, Germany). After the measurements were done, the weight was expressed as relative weight to the body weight of the animal (Chu and Kim 2018). The relative weight (%) was calculated according to the equation: (tissue weight ÷ body weight) × 100.

### Light microscopic observation of epididymal fat cells

Rats were sacrificed to examine epididymal fat tissues, which were removed from the body, fixed with 10% formalin for 48 h, and transferred sequentially to 30%, 50%, 70%, 80%, 90%, 95%, 100% I, and 100% II alcohols for dehydration of the tissues. The tissues were then cleared with xylene and embedded in paraffin. The embedded tissues were cut into 4 μm-thick slices using a microtome. The slices were placed on slide glass, and the paraffin was removed with xylene and then hydrated with graded washes of 100%, 90%, and 80% ethanol. The slices were stained with hematoxylin and eosin, dehydrated, mounted on Canada balsam, and observed via light microscope with a camera (Nikon Eclipse 80i, Japan), with which photos were taken (Chu and Kim 2018).

### Epididymal fat cell size

For measurements of epididymal fat cell size, a microscope with a reticle was used (M309237, ZEISS, Germany) at a low-power field (× 100). Fat cell size was measured 20 times for each group, and the mean was calculated (Chu and Kim 2018).

### Electron microscopic observation

For the electron microscopic observation of hepatocytes, the liver tissues were collected, cut into 1 mm3 samples, and fixed in 2.5% glutaldehyde for two hours. After pre-fixation, the tissue samples were rinsed with a phosphate buffer (4℃, pH 7.4) three times at 10-min intervals and fixed in 1% osmium tetroxide (4℃, phosphate buffer, pH 7.4) for two hours. After post-fixation, the tissues were rinsed three times with the same buffer solution. When the fixation was completed, the tissues were dehydrated with graded series of 50%, 70%, 85%, 90%, 95%, and 100% alcohol, substituted with propylene oxide, and embedded with Epon mixture. The embedded tissues were cut into ultra-thin sections using an ultra-microtome (705,001, Leica Mikrosyteme GmbH, Austria), which were then placed onto the silver grid and double-stained with uranyl acetate and lead citrate for 10 min each and observed via transmission electron microscopy (JEM-210F, JEOL Ltd, Japan) (Chu and Kim 2018).

### Statistical analysis

The experimental results were presented as mean and standard error (Mean ± S.E.). A Student’s t-test was performed to test the differences in the mean among the normal group, control group, and treatment groups, with a *p* value of less than 0.05 being considered statistically significant (Chu and Kim 2018).

## Results

### Body weight measurement and relative weight of liver and epididymal fat

**Fig. 1:**
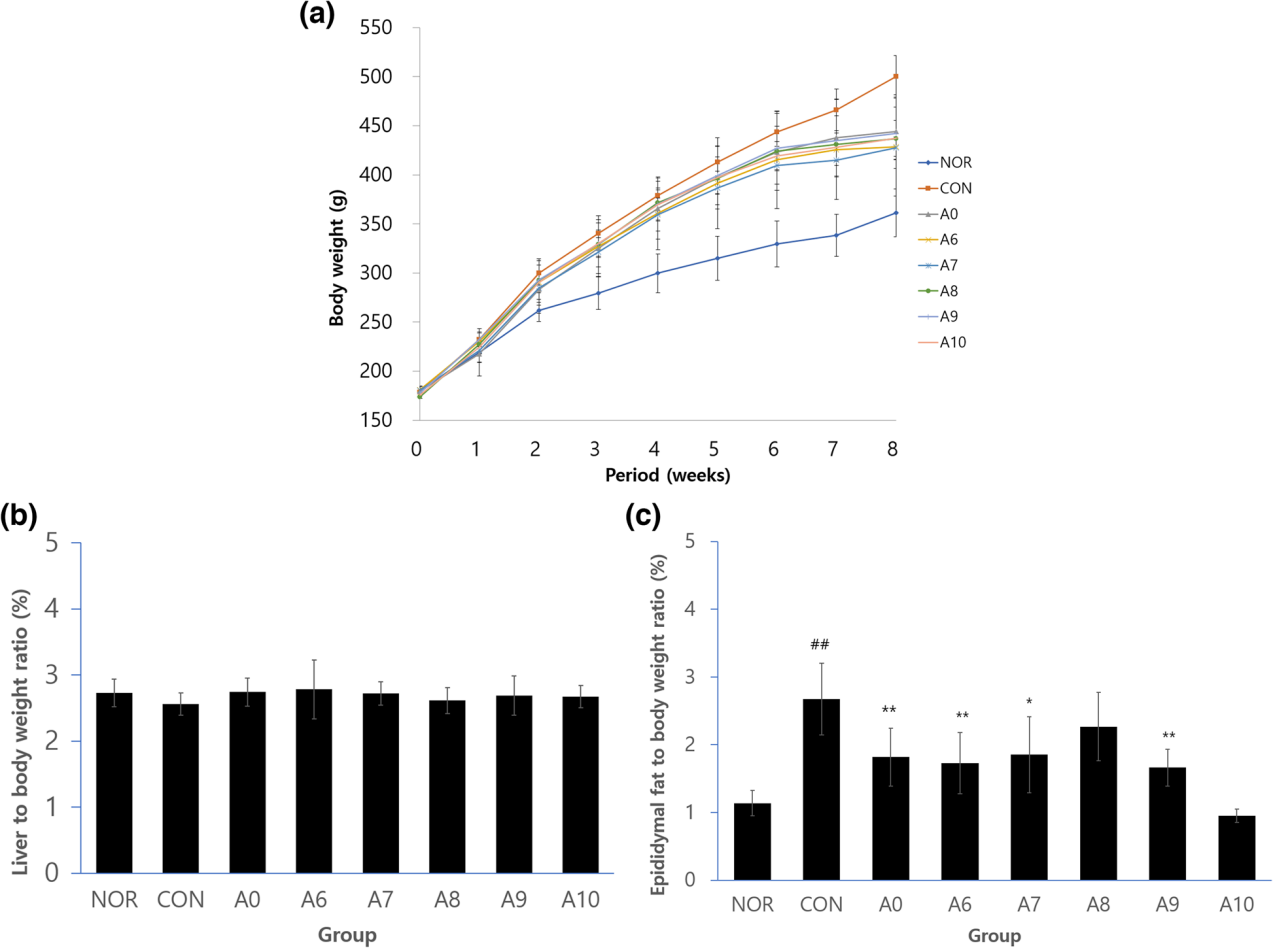
**a** The changes of body weight for 8 weeks. **b** liver to body weight ratio. **c** epididymal fat to body weight ratio. NOR, normal diet group administered water; CON, 60% HFD diet group administered water; A0, 60% HFD diet group treated 2 mL/kg/day *Citrus junos* Sieb ex Tanaka pomace enzyme fermentation extract; A6; A7; A8; A9; A10, 60% HFD diet group treated 2 mL/kg/day *Citrus junos* Sieb ex Tanaka pomace extract fermented with lactic acid bacteria. All values are mean ± S.E.(*n* = 7)

At the initial stage of the experiment, body weight among the normal (NOR), control (CON), and treatment groups was similar, but at the final stage, it was significantly higher in the CON group (500 ± 22 g), by about 28%, compared to the NOR group (361 ± 24 g). The average body weight of the treatment groups (A0, A6, A7, A8, A9, and A10) was significantly lower, by about 13%, than the CON group. The relative weight of the liver was 2.73 ± 0.21% in the normal group and 2.56 ± 0.17% in the CON group. The average relative weight in the treatment groups was 2.70 ± 0.25%, which is not significantly different from the CON group. The relative weight of epididymal fat was 2.67 ± 0.53% in the HFD CON group, which is significantly higher than the 1.14 ± 0.18% of the NOR group. The relative weight of epididymal fat increased significantly in the A0, A6, A7, A9, and A10 groups, to which HFD with fermented yuja extracts were given. In particular, the value for the A10 group was 0.95 ± 0.10%, which is significantly lower than that of the NOR group. The average value of the treatment groups was about 36% lower than that of the CON group (Fig. 1).

### Light microscopic observation of epididymal fat cells

**Fig. 2:**
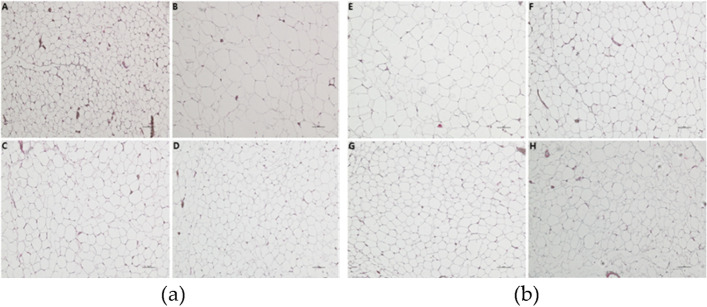
Light micrographs of epididymal fat cell in rats. **a** Group A, normal diet group administered water; Group B, 60% HFD diet group administered water; Group C, 60% HFD diet group administered 2 mL/kg/day *Citrus junos* Sieb ex Tanaka enzyme fermentation extract; Group D, HFD diet group administered 2 mL/kg/day fermented *Citrus junos* Sieb ex Tanaka extract with lactic acid bacteria(*Lactobacillus plantarum* GAVOL-6). **b** Group E–H, HFD diet group administered 2 mL/kg/day fermented *Citrus junos* Sieb ex Tanaka extract with lactic acid bacteria(*Lactobacillus plantarum* GAVOL-7∼10) (Hematoxylin–Eosin stain; X200)

The microscopic observation of epididymal fat tissues showed that the size of the adipocytes was remarkably larger in the CON group (Fig. 2B) compared to the NOR group (Fig. 2A), and the size was slightly larger in treatment groups A0 to A10 (Fig. 2C to Fig. 2H) compared to the NOR group but smaller compared to the CON group.

### Epididymal fat cell size

**Fig. 3:**
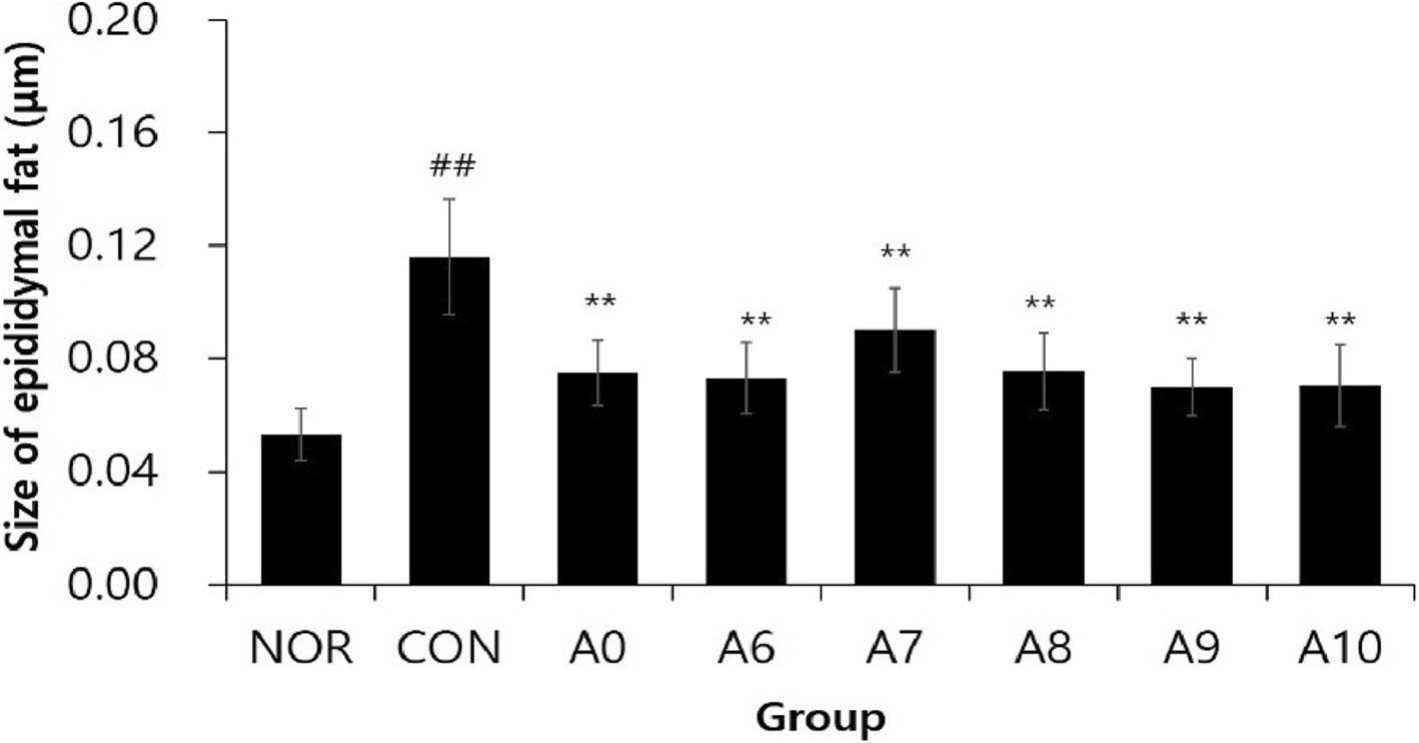
Epididymal fat cell size of rats. NOR, normal diet group administered water; CON, 60% HFD diet group administered water; A0, 60% HFD diet group treated 2 mL/kg/day *Citrus junos* Sieb ex Tanaka pomace enzyme fermentation extract; A6; A7; A8; A9; A10, 60% HFD diet group treated 2 mL/kg/day *Citrus junos* Sieb ex Tanaka pomace extract fermented with lactic acid bacteria. (*n* = 6; ## *p*〈0.01, vs NOR; ** *p*〈0.01, vs CON; × 100)

Epididymal adipocyte size was about 2.4 times larger in the CON group (0.12 ± 0.02 μm) compared to the NOR group (0.05 ± 0.01 μm). The average value of the treatment groups (A6 to A10) given yuja pomace extract fermented with lactic acid bacteria was 0.08 ± 0.01 μm, which was significantly lower, by about 33%, compared to the CON group (*p* < 0.01) (Fig. 3).

### Electron microscopic observation of liver tissues

**Fig. 4:**
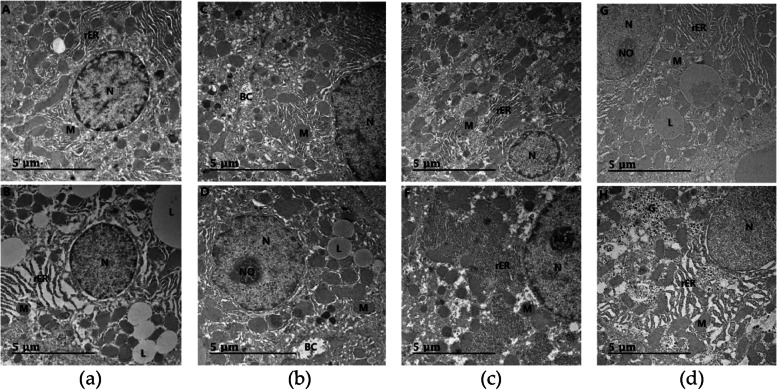
Electron micrographs of hepatocytes of rats. **a** A, normal diet group administered water; B, 60% HFD diet group administered water. A number of lipid droplets are observed in the group B compared with group A. **b** C, 60% HFD diet group treated 2 mL/kg/day *Citrus junos* Sieb ex Tanaka pomace enzyme fermentation extract; D, 60% HFD diet group treated 2 mL/kg/day *Citrus junos* Sieb ex Tanaka pomace extract fermented with lactic acid bacteria(*Lactobacillus plantarum* GAVOL-6). **c** E∼F, 60% HFD diet group treated 2 mL/kg/day *Citrus junos* Sieb ex Tanaka pomace extract fermented with lactic acid bacteria (*Lactobacillus plantarum* GAVOL-7∼8). **d** G∼H, 60% HFD diet group treated 2 mL/kg/day *Citrus junos* Sieb ex Tanaka pomace extract fermented with lactic acid bacteria (*Lactobacillus plantarum* GAVOL-9∼10). L, lipid droplet; M, mitochondria; N, nucleus; NO, nucleolus; rER, rough endoplasmic reticulum; BC, bile canaliculus; G, glycogen (scale bar = 5 μm)

The nuclei of the hepatocytes in the NOR group were round with a relatively even distribution of euchromatin. Many round or elongated mitochondria were observed. The mitochondrial cristae were distinct, and the electron density of the mitochondrial matrix was high. The rough endoplasmic reticula was well developed with stacks of cisternae, and hardly any lipid droplets were observed (Fig. 4A).

The nuclear membranes of the CON group showed a very large nuclear perinuclear space between the inner and outer membranes, and lots of heterochromatin were observed compared to the NOR group. The number of mitochondria was lower than the NOR group, and mostly round mitochondria were observed. The electron density of the mitochondrial matrix was high compared to the NOR group. Rough endoplasmic reticula were observed with stacks of cisternae, as in the NOR group, but the lumen were very dilated. Numerous small and large lipid droplets were observed in the cytoplasm (Fig. 4B).

In the A0 group, which received the enzyme-hydrolyzed yuja pomace extract, the nuclear membrane of the hepatocytes was slightly irregular. Numerous round and elongated mitochondria were observed, as in the NOR group, with distinct mitochondrial cristae. The electron density of the mitochondrial matrix was also similar to that of the NOR group. The rough endoplasmic reticula were very well developed with stacks of cisternae, and lots of lysosomes were observed. Hardly any lipid droplets were observed (Fig. 4C).

In the A6 group, which received the yuja pomace extract fermented with lactic acid bacteria, the nuclear membrane of the hepatocytes was slightly irregular, but the euchromatin was relatively well developed, and a large nucleolus was observed. Numerous round mitochondria were observed. The electron density of the mitochondrial matrix was slightly high, and the rough endoplasmic reticula were highly developed in most parts of the cytoplasm, but did not have stacks of cisternae, as in the NOR group. Lots of lipid droplets were observed (Fig. 4D).

In the A7 group, which received the yuja pomace extract fermented with lactic acid bacteria, the nuclear membrane of the nuclei was round with an even distribution of euchromatin. Numerous round and elongated mitochondria were observed in most parts of the cytoplasm, and the rough endoplasmic reticula were well developed with stacks of cisternae. Hardly any lipid droplets were observed (Fig. 4E).

In the A8 group, which received the yuja pomace extract fermented with lactic acid bacteria, the nuclear membrane of the nuclei was round with well-developed euchromatin, and a large nucleolus was observed. Numerous mitochondria were observed, mostly round-shaped, and the electron density of the mitochondrial matrix was slightly high. The rough endoplasmic reticulum was highly developed, with distinct attached ribosomes. Hardly any lipid droplets were observed (Fig. 4F).

In the A9 group, which received the yuja pomace extract fermented with lactic acid bacteria, the nucleus of hepatocytes was round, and the nucleolus was observed, with an even distribution of euchromatin in the cytoplasm. Numerous round and elongated mitochondria were observed, with distinct mitochondrial cristae. A few lipid droplets were observed, and the endoplasmic reticula were well developed, with stacked cisternae and attached ribosomes (Fig. 4G).

In the A10 group, which received the yuja pomace extract fermented with lactic acid bacteria, the nuclei looked round with an even distribution of euchromatin. Numerous mitochondria were observed, and the electron density of the mitochondrial matrix was high. The shape of the mitochondrial cristae was distinct. Also, lots of glycogen were developed, and hardly any lipid droplets were seen. But the lumen of the endoplasmic reticula was dilated, and the arrangement was irregular (Fig. 4H).

## Discussion

This study confirmed the lopolysis and fat-reducing effects of yuja pomace extract fermented with lactic acid bacteria, which was manufactured using yuja pomace, a byproduct of the yuja manufacturing process, and a safe lactic acid bacteria, *Lactobacillus plantarum* GAVOL-6 to 10, in rats with high-fat diet-induced obesity.

The body weight of rats given yuja pomace extract fermented with lactic acid bacteria for eight weeks was about 13% lower than the control group. It is thought that the fermented extract suppressed lipid accumulation and reduced body weight through various components and mechanisms. Previous studies reported similar results, showing that the administration of Citrus aurantium extract decreased body weight in rats (Calapai et al. 1999), and that citrus juice decreased body weight in rats as well (Deyhim et al. 2006). Thus, the anti-obesity effect of yuja has been proven by this study and previous studies, and yuja pomace extract fermented with lactic acid bacteria, which was manufactured using a functional ingredient of yuja, is believed to have a similar anti-obesity effect.

In the measurement of adipocyte size, it is considered that the hypertrophy of adipocytes is suppressed if cell size is decreased when measuring cells within the same area (Rhee et al. 2007). In this study, the size of epididymal fat cells was significantly smaller in the treatment groups that received yuja pomace extract fermented with lactic acid bacteria compared to the control group, confirming that the fermented extract was effective in reducing adipocyte size. These results are consistent with the results of a previous study in which yuja was found to suppress the functions of adipocytes, reduce blood lipid concentration, and control cell differentiation and proliferation via hormonal stimulation (Kim et al. 2016). It is considered that these results are consistent with our results showing reduced adipocyte size in rats.

Electron microscopy is the only technology through which minute and complicated structures (that cannot be discerned with the naked eye) inside the cell can be observed, and the high resolution of electron microscopy provides a wide range of information (Koster and Klumperman 2003). By taking pictures with electron microscopy, structures such as nuclei, mitochondria, endoplasmic reticula, ribosomes, and glycogen can be observed, and data can be analyzed by comparing the normal group and control group according to these structures and their functional abnormalities (Jayakumar et al. 2011). In a previous study, an investigator observed hepatocytes to identify the effects of obesity on fatty liver. The nuclei of the treatment groups, which received fermented extract, looked similar to those of the normal group, euchromatin was observed in the nucleoplasm, rough endoplasmic reticula were well developed in the cytoplasm, and lots of mitochondria were observed with distinct mitochondrial cristae (Chu and Kim 2018). The results of this study showed that there were fewer lipid droplets in hepatocytes in the treatment groups compared to the control group, the shape of the hepatocyte nuclei was round with nucleolus, and euchromatin was evenly distributed in the cytoplasm. The number of mitochondria was higher than the control group, and round and elongated mitochondria with distinct mitochondrial cristae were observed. The rough endoplasmic reticula were well developed with stacks of cisternae and attached ribosomes. Thus, it is confirmed that the nuclei, mitochondria, and rough endoplasmic reticula in hepatocytes had recovered to the normal condition in the treatment groups administered yuja pomace extract fermented with lactic acid bacteria, which is considered to have an anti-obesity effect. It was reported in previous studies that decreased lipid droplets in the cell and development of endoplasmic reticula observed by electron microscopy were effective in preventing non-alcoholic steatohepatitis (Jayakumar et al. 2011), which is in line with the results in this study, in which decreased lipid droplets and distinct development of endoplasmic reticula were observed in groups given the fermented extract. Also, electron microscopic observation of hepatocytes in rats administered with a fat absorption inhibitor, Orlistat, showed an increased number of mitochondria and nuclei with even and distinct nuclear membranes in drug-treated groups, suggesting anti-obesity effects (Youssef 2018). Based this study’s results showing numerous cell organelles with similar shapes, it is believed that yuja pomace extract fermented with lactic acid bacteria has an anti-obesity effect, given the observation of hepatocyte microstructures.

In conclusion, it was confirmed that yuja pomace extract fermented with lactic acid bacteria is effective in reducing body and tissue weight, and fat cell size in rats with high-fat diet-induced obesity, as well as in suppressing adipocyte production. Moreover, by isolating and identifying novel lactic acid bacteria with a high solubilization rate and functionality for preventing deterioration such as browning and taste during the decomposition and fermentation process, yuzu by-products such as yuzu peels can be used as functional food ingredients.

## Conclusions

In this study, yuja pomace extract fermented with lactic acid bacteria was administered to rats, and various experiments were conducted to prove its anti-obesity effects. It is believed that this study will provide the basic data needed to propose the grounds for the effective utilization of yuja byproducts after food processing. The study was limited to an experimental animal model, but the efficacy of the above fermented extract should be further tested using other experimental models. As various academic and clinical paths exist through which the anti-obesity effects of yuja pomace extract fermented with lactic acid bacteria can be verified, more studies should be conducted in the future.

## Data Availability

All data generated or analyzed during this study are included in this article and no datasets were generated or analyzed during the current study.

## Abbreviations

MRS: De Man, Rogosa and Sharpe
CaCO3: Calcium carbonate
K2HPO4: Dipotassium phosphate
NaCl: Sodium chloride
MgSO4: Magnesium sulfate
(NH4)2SO4: Ammonium sulfate
